# Effect of Cultivated Pastures on Soil Bacterial Communities in the Karst Rocky Desertification Area

**DOI:** 10.3389/fmicb.2022.922989

**Published:** 2022-07-28

**Authors:** Shuzhen Song, Kangning Xiong, Yongkuan Chi, Cheng He, Jinzhong Fang, Shuyu He

**Affiliations:** ^1^School of Karst Science, Guizhou Normal University, Guiyang, China; ^2^State Engineering Technology Institute for Karst Desertification Control, Guiyang, China

**Keywords:** cultivated pasture, vegetation restoration, soil properties, soil bacterial community, karst rocky desertification, Illumina sequencing

## Abstract

Soil bacteria play an important role in regulating the process of vegetation restoration in karst ecosystems. However, the effects of vegetation restoration for different cultivated pastures on soil bacterial communities in the karst rocky desertification regions remain unclear. Therefore, we hypothesized that mixed pasture is the most effective for soil bacterial communities among different vegetation restorations. In this study, we systematically studied the soil properties and soil bacterial communities in four vegetation restoration modes [i.e., *Dactylis glomerata* pasture (DG), *Lolium perenne* pasture (LP), *Lolium perenne* + *Trifolium repens* mixed pasture (LT), and natural grassland (NG)] by using 16S rDNA Illumina sequencing, combined with six soil indicators and data models. We found that the vegetation restoration of cultivated pastures can improve the soil nutrient content compared with the natural grassland, especially LT treatment. LT treatment significantly increased the MBC content and Shannon index. The vegetation restoration of cultivated pastures significantly increased the relative abundance of Proteobacteria, but LT treatment significantly decreased the relative abundance of Acidobacteria. Soil pH and MBC significantly correlated with the alpha diversity of soil bacterial. Soil pH and SOC were the main factors that can affect the soil bacterial community. FAPROTAX analysis showed LT treatment significantly decreased the relative abundance of aerobic chemoheterotrophs. The results showed that the bacterial communities were highly beneficial to soil restoration in the LT treatment, and it confirmed our hypothesis. This finding provides a scientific reference for the restoration of degraded ecosystems in karst rocky desertification areas.

## Introduction

Karst is a special topography or landscape that develops on carbonate rocks like limestone, dolomite, or marble (Tang et al., 2019). Karst landscapes are abundant on the earth’s surface, accounting for approximately 15% of the global land (Yuan, 1991), which can provide drinking water for nearly 20–25% of the global population (Ford and Williams, 2007). In China, not only carbonate rocks are widely distributed but also the karst phenomena are common, with various types, forms, and strong development (Jiang et al., 2014). The karst area in southern China with Guizhou as the center reaches 1.24 million km^2^, with the largest area of continuous exposed carbonate rocks and the strongest karst development among the three major karst-concentrated distribution areas in the world (Xiong et al., 2002). The karst parent rock has a slow soil formation rate, shallow soil layers, broken ground, and concentrated rainstorms, especially disturbed by traditional unprotected farming activities, which can easily cause damage to native vegetation, serious soil erosion, and rocky desertification (Xiong et al., 2017; Chen et al., 2020; Hu and Lan, 2020). Karst rocky desertification is a land degradation process that includes severe soil erosion, extensive exposure of bedrocks, sharp decline in soil productivity, and the emergence of desert-like landscapes, which had become the main ecological disaster that seriously hinders the economic development of southwest China (Xiong et al., 2002).

In order to control the rocky desertification, a large number of ecological restoration projects have been carried out in the karst areas of southwest China. The primary task of comprehensive management of rocky desertification is the restoration and reconstruction of vegetation (Li et al., 2003). In the favorable succession of natural vegetation, grass is the pioneer plant for vegetation restoration and improvement of ecological environment (Qi et al., 2013). The Grain for Green program and the establishment of cultivated pastures are an important part of the restoration of degraded ecosystems (Zhao F. et al., 2019). According to statistics, the area of ecological measures led by the establishment of cultivated pasture has exceeded 10,000 km^2^ (Liu et al., 2021; Qiao et al., 2021). The relevant studies have shown that the establishment of cultivated pastures can reduce the risk of soil erosion, protect the soil surface (Momesso et al., 2022), and improve soil quality and nutrient content (Baptistella et al., 2020). At the same time, it can also provide services for the agricultural ecosystem and improve crop yield (Bonner et al., 2020), which is the last barrier to protect the fragile ecological environment and plays an irreplaceable role in the process of vegetation restoration (Chi et al., 2020).

Soil and vegetation are the most sensitive natural elements in the karst environment (Xiong et al., 2002). Soil degradation not only alters plant composition and reduces the aboveground and underground biomass (Peng F. et al., 2020) but also affects soil nutrient cycling and changes the soil bacterial community diversity (Pan et al., 2018). Vegetation restoration has the potential to restore and improve soil quality and soil microbial diversity in the degraded areas, and it is one of the widely used strategies to restore degraded ecosystems globally (Morrien et al., 2017; Dong et al., 2022). As the most active part of soil, soil microbes are widely involved in energy flow and material cycle in ecosystems, and it is the main driving force of geochemical cycles (Crowther et al., 2019). Soil microbes have important effects on plant health, soil productivity, and ecosystem functioning (Connell et al., 2021). As the most numerous microbial taxa, bacteria are the most important decomposers of soil organic matter and litter, and they play an important role in promoting nutrient cycling and regulating ecosystem processes (Yang et al., 2021) and in determining the development direction of degraded ecosystems (Singh, 2015). Soil bacterial communities are extremely sensitive to environmental changes, and small changes in environmental factors can cause changes in their diversity and quantity, so they are often used as indicators of ecosystem changes (Sui et al., 2019). At present, the knowledge of the factors driving the soil microbial community structure and diversity is still limited, so it is of great significance to explore the intrinsic relationship between these two under different vegetation restorations (Wang G. Z. et al., 2021). Therefore, studying the composition and changes of soil bacteria will help us not only better understand the process of ecosystem degradation and restoration but also predict and control the development of degraded ecosystems (Sun et al., 2022).

The karst area of southwest China is one of the typical fragile ecosystems in the world with a fragile ecological environment, serious rocky desertification, and prominent contradiction between human and land (Wang et al., 2019). Soil function restoration in the karst areas is crucial for the regeneration of the degraded ecosystems (Guo et al., 2019), and current research in this area mainly focuses on the impact of vegetation succession on soil function. For example, Li et al. (2021) studied the changes of soil microbial communities during the succession of vegetation from bare rock to arbor forest. Mi et al. (2021) studied soil genetic diversity of bacteria and fungi in different vegetation successions (grassland, shrub, natural forest, and secondary forest) in karst areas. Wang G. Z. et al. (2021) explored the effects of secondary succession on the composition and diversity of soil fungi and bacteria in karst areas. However, there less research that has been done on the restoration of artificial vegetation in karst areas (Lu et al., 2022). Furthermore, few studies have focused on bacterial communities and environmental factors under several different artificial vegetation restorations (Chen et al., 2020). At present, the difference between the natural restoration and artificial grass planting of fragile ecosystems in the karst rocky desertification control area in southwest China is unclear, and also the differences in soil microbial communities between different restorations of cultivated pastures are unclear.

In this study, we aimed to (1) investigate changes in soil chemical indices and microbial biomass after 10-year vegetation restoration in different cultivated pastures; (2) assess the effects of different vegetation restorations on soil bacterial structure and diversity; and (3) determine the relationship between soil bacterial communities and environmental factors. We hypothesized that mixed pastures had the most effective on soil bacterial communities among vegetation restorations. To test this hypothesis, we investigated the soil properties and soil bacterial communities in four vegetation restoration modes [i.e., *Dactylis glomerata* pasture (DG), *Lolium perenne* pasture (LP), *Lolium perenne* + *Trifolium repens* mixed pasture (LT), and natural grassland (NG)] by using 16S rDNA Illumina sequencing, combined with six soil indicators and data models. We found that LT treatment can significantly improve the soil nutrient content and soil microbial biomass, significantly increase the relative abundance of Proteobacteria, but significantly decrease the relative abundance of Acidobacteria and aerobic chemoheterotrophs compared with other vegetation restoration modes. This study provided a scientific basis for the vegetation restoration management of karst-degraded ecosystems in southwest China.

## Materials and Methods

### Study Area and Sampling

The study area is a demonstration area for the karst rocky desertification control in Salaxi Town, Bijie city, Guizhou Province, China (105^°^02′01′′-105^°^08′09′′E, 27^°^11′36′′-27^°^16′51′′N), belonging to a typical light-to-moderate rocky desertification area, with an average altitude of 1,600 m. The area has a mid-subtropical monsoon climate, an annual average temperature of 12^°^C, a frost-free period of 245 days, an annual average sunshine duration of 1,360 h, and an average annual rainfall of 984.4 mm. The seasonal distribution of precipitation is uneven, with more than 80% of the precipitation concentrated in June to September. The soil is developed from limestone, and the main soil type is yellow soil. The vegetation is dominated by *Cyclobalanopsis glauca*, *Pyracantha fortuneana*, *Rhododendron simsii*, and *Pinus yunnanensis*. The rocky desertification area is 55.931 km^2^ in the study area, accounting for 64.93% of the total area of the demonstration area. In order to control rocky desertification, the research group cultivated pasture in the study area in 2012. The pastures were planted mainly including perennial ryegrass (*Lolium perenne*), white clover (*Trifolium repens*), and orchardgrass (*Dactylis glomerata*). The methods of cultivated pasture were single and mixed sowing. In addition to the restoration with cultivated pasture, there was also a restoration method of natural grassland. Natural grassland mainly included *Artemisia lavandulaefolia*, *Chenopodium glaucum*, *Clinopodium chinense*, *Plantago asiatica*, *Stellaria media*, *Digitaria sanguinalis*, and *Polygonum hydropiper*.

In mid-April 2021, the *Dactylis glomerata* pasture (DG), *Lolium perenne* pasture (LP), *Lolium perenne* + *Trifolium repens* mixed pastures (LT), and natural grassland (NG) with the same site conditions were selected as the test sample plots in the study area, and the natural grassland (NG) as a control. Each test plot was set up with six sampling plots of 10 m × 10 m (24 sampling plots in total), and the distance between plots and the boundary of the plot was greater than 10 m. In each sampling plot, the S-shaped multi-point sampling method was used, and 15 sampling points were evenly set (the sampling points were about 3 cm away from the base of the plant). After removing the litter layer on the soil surface, soil samples at the surface (0–10 cm) were collected with a soil drill. To avoid spatial heterogeneity, soil samples from 15 sampling points were mixed into one replicate, and a total of 24 soil samples were obtained. After removing impurities from the soil samples, which were divided into two parts: one part was placed in sterilized 15-ml centrifuge tubes, stored in liquid nitrogen, and transported back to the laboratory, where these samples were stored in a −80^°^C freezer for 16S rDNA analysis; another part of the soil samples was air-dried indoors and then passed through a 2-mm sieve for the determination of soil properties.

### Determination of Soil Property

Soil pH was determined by using a glass electrode using a 1:2.5 soil/water mixture, soil organic carbon (SOC) was determined by using the potassium dichromate oxidation–external heating method, total nitrogen (TN) was determined by using the sulfuric acid catalyst digestion–Kjeldahl method, and total phosphorus (TP) was determined by using the concentrated sulfuric acid digestion–Mo-Sb colorimetric method (Bao, 2000), and then the continuous flow analyzer (SYSTEA, FLOWSYS, Italy) was used to determine soil TN and TP. The chloroform fumigation–extraction method (Lin, 2010) and the elemental analyzer (FlashSmart, Thermo Fisher, United States) were used to determine the soil microbial biomass carbon (MBC) and microbial biomass nitrogen (MBN).

### DNA Extraction and Amplicon Sequencing

DNA was extracted using the TGuide S96 Magnetic Soil DNA Kit [Tiangen Biotech (Beijing) Co., Ltd.] according to the manufacturer’s instructions. The DNA concentration of the samples was measured using the Qubit dsDNA HS Assay Kit and Qubit 4.0 Fluorometer (Invitrogen, Thermo Fisher Scientific, Oregon, United States). The 338F: 5′-ACTCCTACGGGAGGCAGCA-3′ and 806R: 5′-GGACTACHVGGGTWTCTAAT-3′ universal primer set was used to amplify the V3-V4 region of 16S rRNA gene from the genomic DNA extracted from each sample. Both the forward and reverse 16S primers were tailed with sample-specific Illumina index sequences to allow for deep sequencing. The PCR was performed in a total reaction volume of 10 μl: DNA template 5–50 ng, *Vn F (10 μM) 0.3 μl, *Vn R (10 μM) 0.3 μl, KOD FX Neo Buffer 5 μl, dNTP (2 mM each) 2 μl, KOD FX Neo 0.2 μl, and ddH_2_O up to 10 μl. Vn F and Vn R were selected according to the amplification area. After that, the following steps were carried out: initial denaturation at 95^°^C for 5 min, followed by 25 cycles of denaturation at 95^°^C for 30 s, annealing at 50^°^C for 30 s, and extension at 72^°^C for 40 s, and a final step at 72^°^C for 7 min. The total of PCR amplicons were purified with Agencourt AMPure XP Beads (Beckman Coulter, Indianapolis, IN) and quantified using the Qubit dsDNA HS Assay Kit and Qubit 4.0 Fluorometer (Invitrogen, Thermo Fisher Scientific, Oregon, United States). After the individual quantification step, amplicons were pooled in equal amounts. For the constructed library, Illumina NovaSeq 6000 (Illumina, Santiago CA, United States) was used for sequencing.

### Sequence Analysis

The bioinformatics analysis of this study was performed with the aid of the BMK Cloud (Biomarker Technologies Co., Ltd., Beijing, China). According to the quality of single nucleotides, raw data were primarily filtered by Trimmomatic (version 0.33) (Edgar, 2013). Identification and removal of primer sequences were processed by Cutadapt (version 1.9.1) (Callahan et al., 2016). PE reads obtained from previous steps were assembled by USEARCH (version 10) (Segata et al., 2011), followed by chimera removal using UCHIME (version 8.1) (Quast et al., 2013). The high-quality reads generated from the aforementioned steps were used in the following analysis. Sequences with a similarity ≥ 97% were clustered into the same operational taxonomic unit (OTU) by USEARCH (v10.0) (Edgar, 2013), and the OTUs with re-abundance < 0.005% were filtered. Taxonomy annotation of the OTUs was performed based on the Naive Bayes classifier in QIIME2 (Bolyen et al., 2019) using the SILVA database (release 132) (Quast et al., 2013) with a confidence threshold of 70%. The alpha diversity was calculated and displayed by QIIME2 and R software, respectively. Beta diversity was determined to evaluate the degree of similarity of microbial communities from different samples using QIIME.

### Statistical Analysis

The taxonomic alpha diversity (i.e., Shannon–Wiener index, abundance-based coverage estimators (ACE), and Chao 1) was analyzed by QIIME2 software. The shared and unique OTUs among samples were used to generate Venn diagrams. Differences in soil properties, bacterial diversity indices, and differential OTUs (relative abundance > 1%) among different vegetation restorations were tested by one-way analysis of variance (ANOVA), multiple comparisons (Duncan’s multiple range test), and the least significant difference by using IBM SPSS Statistics 19.0 (version 19.0; SPSS, Chicago, IL, United States). Principal coordinate analysis (PCoA) was analyzed the beta diversity between different samples based on the Bray–Curtis distance matrix by using QIIME, and the analysis of the dissimilar test was carried out in ANOSIM. The relative abundance of bacterial phylum and ecological function prediction of soil bacterial community was converted from the percentages to a log series to fulfill normality of residuals. The Pearson correlation analysis method was used to analyze the correlation between environmental factors and alpha diversity of soil bacterial community. Redundancy analysis (RDA) based on forward selection was used to select the explanatory environmental factors that had a significant (*P* < 0.05) impact on the variation of the soil bacterial community. The analysis of the Monte Carlo permutation test was performed to test the correlation of environmental factors with bacterial community composition by using Canoco (version 5.0 for Windows; Ithaca, NY, United States). Functional Annotation of Prokaryotic Taxa database 12 (FAPROTAX, v1.2.3) was used to predict ecologically relevant functions of bacteria derived from 16S rRNA amplicon sequencing, which is widely used for biogeochemical cycling processes in environmental samples (especially carbon, hydrogen, and element cycles such as nitrogen, phosphorus, and sulfur) for functional annotation prediction (Sansupa et al., 2021).

## Results

### Soil Properties and Soil Microbial Biomass Under Different Vegetation Restorations

**FIGURE 1:**
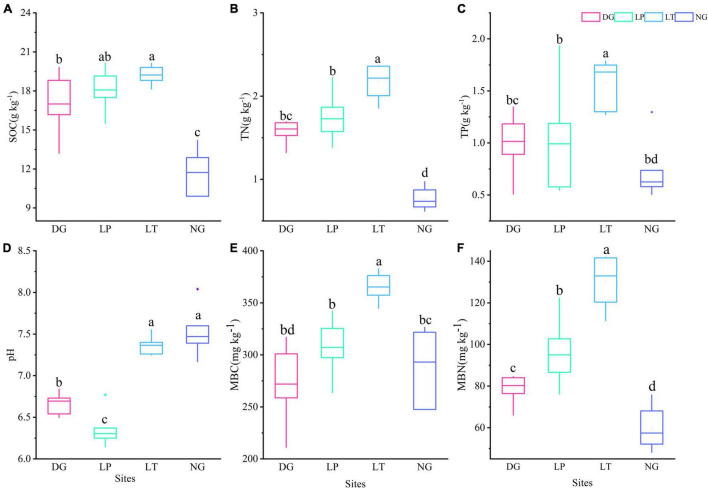
Soil properties and soil microbial biomass in different vegetation restorations. Boxplots with different lowercase letters are significantly different among different sites (*p* < 0.05). **(A)** The content of SOC; **(B)** the content of TN; **(C)** the content of TP; **(D)** pH value; **(E)** the content of MBC; **(F)** the content of MBN. DG, *Dactylis glomerata*; LP, *Lolium perenne*; LT, *Trifolium repens*; NG, natural grassland; SOC, soil organic carbon; TN, total nitrogen; TP, total phosphorus; MBC, soil microbial biomass C; MBN, soil microbial biomass N.

In the 10-year vegetation restoration, soil properties changed significantly (*P* < 0.05) under different vegetation restoration treatments (Figures 1A–D). The SOC content varied from 9.9 to 20.13 g.kg^–1^. The LT treatment had a more concentrated distribution of SOC, while DG treatment had the largest variance. Compared with the control group (NG), the SOC content increased significantly in the DG, LP, and LT treatments (*P* < 0.05), but there was no significant difference between DG and LP treatments, and there was also no significant difference between the LP and LT treatments. The median content of TN varied from 0.74 to 2.22 g.kg^–1^. The TN content increased significantly in LT treatment (*P* < 0.05), but there was no significant difference between DG and LP treatments. The TP content varied from 0.51 to 1.93 g.kg^–1^. The TP content in LT treatment was significantly higher than that in other treatments (*P* < 0.05), but there was no significant difference between DG, LP, and NG treatments. The median content of pH values in our study area ranged from 6.31 to 7.47. The pH values was significantly decreased in the DG and LP treatments (*P* < 0.05), and it was lowest in the LP treatment.

There were significant differences (*P* < 0.05) in soil MBC and MBN among different vegetation restoration treatments (Figures 1E,F). The median content of soil MBC and MBN was highest in LT treatment. The soil MBC content ranged from 211.20 to 382.47 mg.kg^–1^. The LT treatment had a more concentrated distribution of soil MBC, while DG treatment had the largest variance. The median content of soil MBN varied from 57.45 to 132.93 mg.kg^–1^. The DG treatment had a more concentrated distribution of soil MBN, while LT treatment had the largest variance. The soil MBN content from highest to lowest were for LT, LP, DG, and NG treatments.

### Soil Bacterial Composition and Diversity Under Different Vegetation Restorations

A total of 1,919,684 raw reads were detected in all samples, generating an average of 79,561 clean reads and an average sequence length of 416.33 bp (Supplementary Table 1). The species composition of all samples included 28 phyla, 58 classes, 159 orders, 292 families, 518 genera, and 589 species. When the distance level was 0.03, the rarefaction curve of bacterial communities in different vegetation restoration treatments reached asymptotes (Supplementary Figure 1), which indicated that the diversity of soil bacterial communities was well extracted. We illustrated the similarities and differences among OTUs of different treatments in three 2-set Venn diagrams (Supplementary Figures 2–4). The unique OTUs in the DG and NG treatments were 93 and 192, respectively, and the shared OTU was 2,112 (Supplementary Figure 2). The unique OTUs in the LP and NG treatments were 79 and 350, respectively, and the shared OTU was 1,954 (Supplementary Figure 3). The unique OTUs in the LT and NG treatments were 155 and 100, respectively, and the shared OTU was 2,204 (Supplementary Figure 4).

**FIGURE 2:**
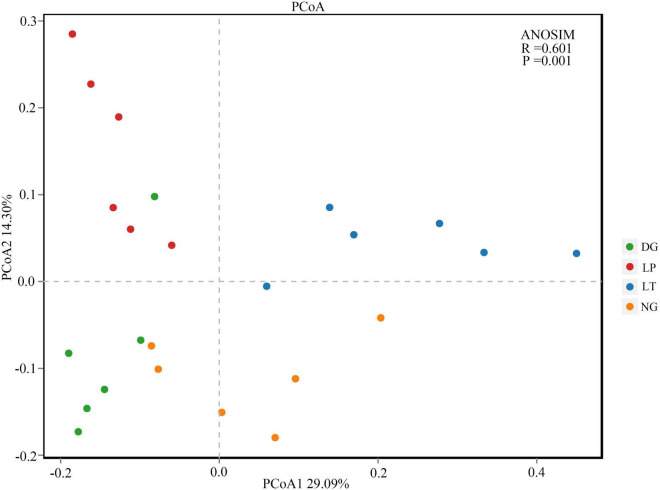
Principal coordinates analysis of bacterial in different vegetation restorations. DG, *Dactylis glomerata*; LP, *Lolium perenne*; LT, *Trifolium repens*; NG, natural grassland.

**FIGURE 3:**
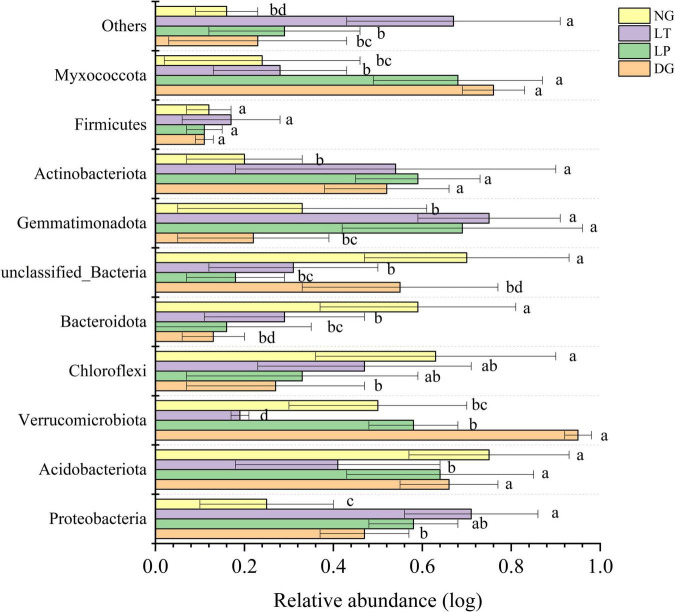
Relative abundance of soil bacterial phylum in different vegetation restorations. Different lowercase letters are significantly different among different sites (*p* < 0.05). DG, *Dactylis glomerata*; LP, *Lolium perenne*; LT, *Trifolium repens*; NG, natural grassland.

The results of PCoA showed that the dissimilar test in ANOSIM had significant differences between groups (*R* = 0.601, *P* = 0.001), and the bacterial communities of different treatments were separated (Figure 2), which indicated that the vegetation restoration treatment resulted in a significantly changed bacterial community structure. Among the top 10 bacterial phyla in relative abundance of the bacterial community in vegetation restoration treatment (Figure 3), Proteobacteria and Acidobacteriota were the dominant bacterial phyla, accounting for 63.27% of the total bacterial abundance. The results showed that compared with NG treatment, LT treatment significantly increased the relative abundance of Proteobacteria, Gemmatimonadota, and Actinobacteriota (*P* < 0.05) but significantly decreased the relative abundance of Acidobacteriota, Verrucomicrobiota, and Bacteroidota (*P* < 0.05).

We performed multiple comparisons of Alpha diversity in different vegetation restoration treatments (Table 1). The Shannon index in the LT treatment was significantly higher than that in DG, LP, and NG treatments (*P* < 0.05), but there was no significant difference between DG, LP, and NG treatments. The ACE index and Chao1 index in the DG treatment were significantly lower than that in LP, LT, and NG treatments (*P* < 0.05), but there was no significant difference between the LP, LT, and NG treatments.

**TABLE 1:** Alpha diversity analysis of bacterial in different vegetation restorations.

	Shannon	ACE	Chao1
DG	8.37 ± 0.22*bd*	1603.06 ± 59.03*b*	1638.29 ± 54.60*b*
LP	8.48 ± 0.39*bc*	1820.21 ± 104.41*a*	1853.40 ± 91.31*a*
LT	9.02 ± 0.23*a*	1935.50 ± 79.38*a*	1971.28 ± 84.11*a*
NG	8.60 ± 0.21*b*	1898.55 ± 152.50*a*	1944.28 ± 132.56*a*
*F-*value	7.061**	12.174**	15.221**

*Data are mean ± standard error. Different lowercase letters in the same column indicate significant difference at 0.05 levels. The F value is the statistic for the F-test.*

**Significant at the 0.01 probability level.

*DG, Dactylis glomerata; LP, Lolium perenne; LT, Trifolium repens; NG, natural grassland.*

### Effects of Environmental Factors on Soil Bacterial Community

We performed a correlation analysis of soil properties and alpha diversity of soil bacterial microbial communities (Table 2). The results showed that the Shannon index, ACE index, and Chao1 index had a significant positive correlation with pH and MBC, which indicated that soil pH and MBC were important factors to affect the alpha diversity of soil microbial community. The correlation analysis of soil microbial biomass and soil properties showed that MBC and MBN had a significant positive correlation with SOC, TN, and TP.

**TABLE 2:** Correlation between alpha diversity of soil bacterial communities and microbial biomass and soil properties.

	pH	SOC	TN	TP	MBC	MBN
Shannon	0.661**	–0.028	0.051	–0.009	0.487*	0.275
ACE	0.439*	–0.003	–0.043	0.372	0.567**	0.195
Chao1	0.479*	–0.032	–0.060	0.346	0.570**	0.191
MBC	0.186	0.682**	0.547**	0.611**	/	0.700**
MBN	–0.057	0.735**	0.943**	0.663**	0.700**	/

*Correlation was significant at the 0.05 level (two-tailed). **Correlation was significant at the 0.01 level (two-tailed). SOC, soil organic carbon; TN, total nitrogen; TP, total phosphorus; MBC, soil microbial biomass C; MBN, soil microbial biomass N.

**FIGURE 4:**
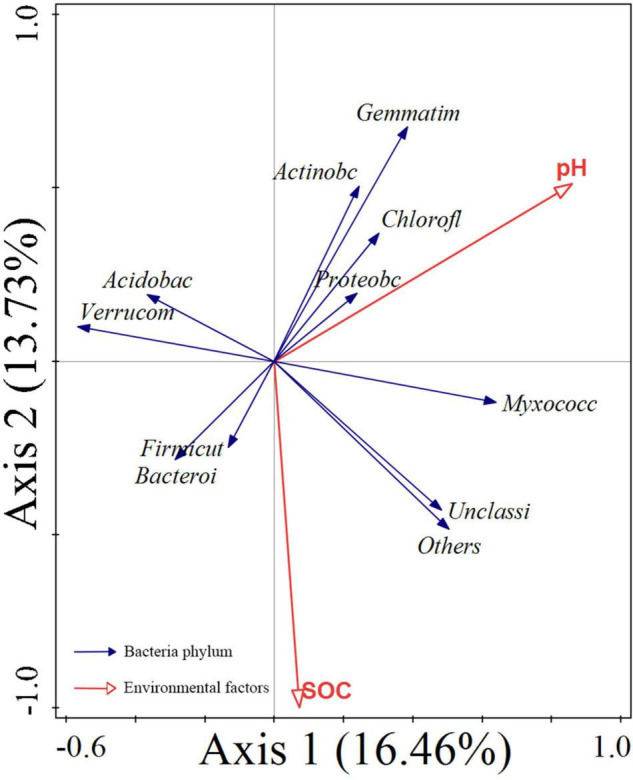
RDA for the relationship between the bacterial phylum (blue arrows) and environmental factors (red arrows). SOC, soil organic carbon; *Proteoba*, Proteobacteria; *Acidobac*, Acidobacteriota; *Verrucom*, Verrucomicrobiota; *Chlorofl*, Chloroflexi; *unclassi*, unclassified Bacteria; *Bacteroi*, Bacteroidota; *Gemmatim*, Gemmatimonadota; *Actinobc*, Actinobacteriota; *Myxococc*, Myxococcota; *Firmicut*, Firmicutes.

Redundancy analysis of the soil microbial structure at the phylum level with soil properties showed that the first and second axes explained 16.46 and 13.73% of bacterial community variation, respectively (Figure 4). Further analysis by using the Monte Carlo permutation test showed that pH and SOC had higher correlations with bacterial community composition than other environmental factors, contributing 33.4% (*p* = 0.002) and 30.6% (*p* = 0.002), respectively, which suggested that they were the main factors affecting the bacterial community structure. Soil pH was positively correlated with Proteobacteria, Chloroflexi, Gemmatimonadota, Actinobacteriota, and Myxococcota. SOC was positively correlated with Bacteroidetes, Firmicutes, and Myxococcota. Soil pH and SOC were negatively correlated with Acidobacteriota.

**FIGURE 5:**
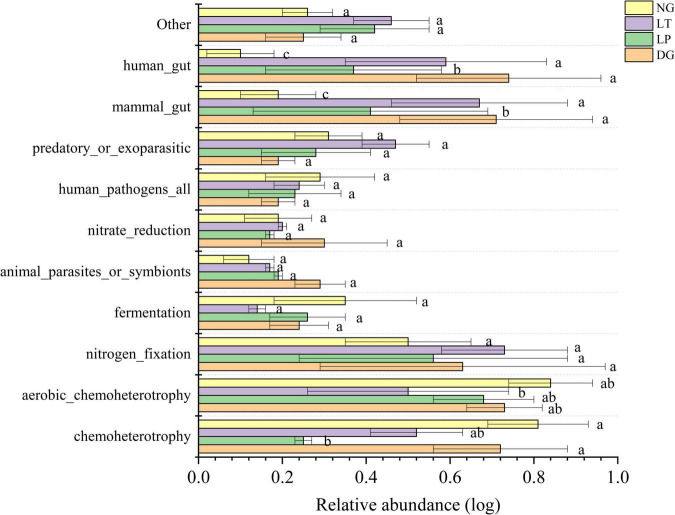
Relative abundance of soil bacterial community for ecological function in different vegetation restorations. Different lowercase letters are significantly different among different sites (*p* < 0.05). DG, *Dactylis glomerata*; LP, *Lolium perenne*; LT, *Trifolium repens*; NG, natural grassland.

The ecological function prediction of soil bacteria in different vegetation restoration treatments was performed by FAPROTAX software (Figure 5). Among all ecological function groups, the average relative abundances of chemoheterotrophs, aerobic chemoheterotrophs, nitrogen fixation, and fermentation were 30.82, 25.02, 6.79, and 4.48%, respectively. The results showed that the relative abundance of aerobic chemoheterotrophs in the LT treatment was significant lower than that in DG, LP, and NG treatments (*P* < 0.05), but there was no significant difference between DG, LP, and NG treatments. The relative abundance of chemoheterotrophs, nitrogen fixation, and fermentation in the DG, LP, LT, and NG treatments was not significant.

## Discussion

### Effects of Different Vegetation Restorations on Soil Properties and Soil Microbial Biomass

The goal of vegetation restoration was not only the increase in vegetation coverage but also the restoration of ecosystem quality and function (Wang et al., 2020). The vegetation restoration of cultivated pasture can improve soil quality and nutrient content and effectively prevent soil degradation (Baptistella et al., 2020). After 10 years of vegetation restoration, there were significant differences in soil properties under different vegetation restoration treatments. The related studies have shown that converting land use from agriculture to cultivated pasture reduced soil acidity (Chen et al., 2020), which was consistent with the findings in this study that DG, LP, and LT treatments reduced soil pH. This may be because cultivated pasture has higher aboveground biomass, which can produce a large amount of easily decomposable litter, increase the community of burrowing animals, and accelerate the cation cycle in the grassland ecosystem, thereby offsetting soil acidification (Paudel et al., 2021). In this study, DG, LP, and LT can significantly increase the SOC content compared to NG, which was consistent with the findings of Zhang et al. (2019) and Peng X. D. et al. (2020); this may be due to the increase of surface litter and underground roots in cultivated pasture, which increases the source of soil organic carbon and promotes the accumulation of organic carbon (Wu Q. et al., 2019). DG and LP significantly increased the TN content, and the TN content in the LT treatment was the highest. The relevant studies have shown that mixed pasture was more conducive to the accumulation of soil total nitrogen (Yang et al., 2013). In addition, the biological nitrogen fixation of white clover in the LT treatment increased the nitrogen content (Young et al., 2019), and Khatiwada et al. (2020) believed that mixed pasture was relatively stable, with strong root activity, improved photosynthetic capacity, and strong soil microbial activity, which can improve nitrogen accumulation capacity. However, the total nitrogen content of NG was 0.94 ± 0.02 g.kg^–1^ in our study. N was the factor limiting the growth of natural grassland according to the second national soil nutrient classification standards. The result of this study was inconsistent with that of Xing et al. (2020), which may be caused by differences in the study area, grass species, and climate conditions. Relevant studies have shown that the establishment of cultivated pasture can improve grassland productivity and bring more organic matter to the topsoil (Lin et al., 2019), and the main direct source of phosphorus was the decomposition of soil organic matter (Donald, 1980). In this study, DG, LP, and LT treatments increased the TN content, especially LT, which may be because the mixed pasture can form the spatial advantage of phosphorus nutrient utilization and the difference in phosphorus source utilization in the rhizosphere, and ultimately promote mutual absorption and improve the soil phosphorus content (Guerra et al., 2022).

Soil microbes are exceptionally sensitive to changes in the environment, and microbial biomass plays a key role in organic matter dynamics and nutrient cycling in the lithosphere (Pang et al., 2018). The maintenance of soil microbial life needs to consume some energy, and litter is one of the sources of energy raw materials for soil microorganisms, so the input of litter can change the soil microbial biomass on the soil surface (Lu et al., 2022). In this study, LT treatment significantly increased soil MBC and MBN, which was mainly associated with the large input of surface litter from mixed pasture, and it can provide sufficient energy for soil microbial metabolism and self-synthesis (Grigera and Oesterheld, 2021). In addition, the soil MBC content of DG treatment was slightly lower than that of NG treatment, but there was no significant difference between DG and NG treatments, which may be that the diversity of litter also affected soil microbial biomass carbon (Hu et al., 2006). The SOC content in the LT treatment in this study was significantly higher than that in NG, and both soil MBC content and soil MBN content also showed the same trend, which was consistent with the results of Kan et al. (2022). In this study, the soil MBN content in the DG, LP, and LT treatments was significantly higher than that in NG, which may be because cultivated pasture planting increased the soil TN content, thereby increasing soil MBN, but the internal mechanism needs to be further studied.

### Effects of Different Grassland Vegetation Restorations on Soil Microbial Structure and Diversity

The structure and diversity of soil microorganisms have important effects on the stability of karst ecosystems (Xie et al., 2015). The PCoA results showed that the dissimilar test in ANOSIM showed significant differences between groups, and the bacterial communities of different treatments were separated (Figure 2), which indicated that the different vegetation restoration treatments significantly changed the bacterial community structure. In this study, Proteobacteria and Acidobacteriota were the dominant phyla (Figure 3), which was consistent with the findings in different soil environments (Gao et al., 2017; Zhang et al., 2021; Zhu et al., 2021). In the existing reports, Proteobacteria was considered to be the most common bacterial phylum in the world (Wu W. X. et al., 2019). Vegetation restoration can increase the relative abundance of Proteobacteria because soil organic carbon contributes to the survival and development of Proteobacteria (Zeng et al., 2017). Different vegetation restoration of cultivated pasture in this study increased the soil organic carbon content, which indicated that Proteobacteria was promoted by soil organic carbon (Hartman et al., 2008), and cultivated pasture provided a better development environment for the development of Proteobacteria. Acidobacteriota was closely related to soil nutrition; generally speaking, the abundance of Acidobacteriota was higher in nutrient-poor soils, and they can be used as indicator bacteria of poor soil environment (Fierer et al., 2007). In this study, the LT treatment decreased the relative abundance of Acidobacteriota, which indicated that vegetation restoration improved the soil environment and quality.

The diversity of soil microorganisms reflected the stability of the microbial community and the influence of the soil environment on its community structure, and it was a key indicator of soil ecological characteristics (Li et al., 2020). In this study, the diversity indices (Shannon, ACE, and Chao1) of the soil bacterial microbial community in the DG treatment were lower than those in other treatments, but they were most abundant in the LT treatment. This may be because the response of microbial diversity to changes in aboveground vegetation was mainly affected by changes in the relative abundance of species, rather than changes in species diversity (Friedman et al., 2017). Soil analysis showed that the soil quality in the LT treatment was higher than that in the NG, indicating that the mixed pasture could create a suitable microenvironment for the survival of bacteria and also improve the soil bacterial community diversity index.

### Effects of Environmental Factors on Soil Bacterial Community in Different Vegetation Restorations

The Shannon index, ACE index, and Chao1 index had a significant positive correlation with pH and MBC from Pearson correlation analysis (*P* < 0.05), which indicated that soil pH and MBC had significant effects on the alpha diversity of soil bacteria in different vegetation restoration treatments. This may be because vegetation restoration balanced the pH of the soil and improved the living environment of soil microorganisms, which confirms that vegetation restorations can improve the environment and improve soil quality (Zhao C. et al., 2019). Soil microbial biomass was extremely sensitive to changes in aboveground vegetation and belowground conditions, and it can be used as a sensitive factor for changes in soil quality (Yin et al., 2014). In this study, SOC, TN, and TP significantly increased the content of soil MBC and MBN.

Numerous studies have shown that the soil bacterial community structure was affected by environmental conditions, and soil factors were the key influencing factors (Saleem, 2015; Chen et al., 2017). In this study, RDA results indicated that pH in different vegetation restorations was one of the key factors affecting soil microbial communities, which was consistent with the findings of Fan et al. (2019) and Wang Z. Q. et al. (2021). It has been confirmed in many studies that soil pH was a key factor affecting the soil microbial community structure (Griffiths et al., 2011; Ling et al., 2016; Yun et al., 2016; Zhang et al., 2022). Furthermore, our results showed that the relative abundance of Actinobacteria was significantly affected by soil pH, which was consistent with the findings of Tripathi et al. (2012) and Wang Z. Q. et al. (2021). The strong correlation between pH and the soil microbial community structure may be due to the relatively narrow growth tolerance and a harsh growth environment exhibited by most bacterial taxa in karst environments (Xue et al., 2017). In our study, it was found that pH was negatively correlated with Acidobacteriota, which was inconsistent with the results of many studies (Dai et al., 2017; Zhang et al., 2020), which indicated that Acidobacteriota of different land use types was closely related to pH. In this study, Proteobacteria was the dominant phylum, and previous studies have shown that Proteobacteria was positively correlated with pH of the soil (Zhang et al., 2021), which was consistent with our findings. Soil organic carbon was an important factor affecting the soil bacterial community structure, which was consistent with the research results of Shao et al. (2005) and Xue et al. (2019), that is, soil organic carbon was an important indicator affecting the soil microbial community structure.

Soil microbial functional diversity was the guarantee of soil function and the basis for restoring soil function (Zi et al., 2017). Chemoheterotrophic bacteria mainly consume organic matter for energy, and fermentative bacteria can metabolize carbohydrates into lactic acid (Ling et al., 2022). Among agricultural soil bacteria, it was mainly chemical heterotrophic bacteria that can degrade aromatic compounds and aliphatic hydrocarbons (Qi et al., 2018). However, grassland soil bacteria have less chemical heterotrophic bacteria than agricultural soil bacteria (Zhu et al., 2022). The results of soil bacterial ecological function prediction showed that the relative abundance of aerobic chemoheterotrophic bacteria was the highest in the NG treatment and the lowest in the LT treatment; this may be because a large amount of litter in the NG treatment was not easily utilized by plants, resulting in a large number of chemoheterotrophic bacterial groups, but the litter could be decomposed in the LT treatment in time, thus reducing the abundance of chemical heterotrophic bacteria. The result indicated that LT treatment brought soil bacterial function closer to grassland.

## Conclusion

This study took the karst ecosystem in southwest China as the research object. After 10 years of vegetation restoration, we determined the effects of cultivated pasture on soil properties, soil microbial biomass, and bacterial communities. We concluded that the three vegetation restorations of DG, LP, and LT significantly increased the relative abundance of Proteobacteria, but LT treatment obviously decreased the relative abundance of Acidobacteriota. The bacterial community in the vegetation restoration of cultivated pasture improved the soil environment; increased the contents of SOC, TN, and TP; and promoted the soil to develop in a good direction, especially the LT treatment. LT treatment significantly increased the MBC content and Shannon index but significantly decreased the relative abundance of aerobic chemoheterotrophs. Soil pH and MBC had a significant positive correlation with the Shannon, ACE, and Chao1 indices. Soil pH and SOC were the main factors that can affect soil bacterial communities. Therefore, cultivating LT in the process of vegetation restoration is an effective measure to promote soil restoration in karst areas.

## Data Availability Statement

The datasets presented in this study can be found in online repositories. The names of the repository/repositories and accession number(s) can be found below: NCBI BioProject–PRJNA842166.

## Author Contributions

SS performed the sampling and contributed to the experimental design, data analysis, and writing of the manuscript. YC and CH contributed to the sampling and data analysis. KX and JF provided to the study site. SS and SH contributed to the data analysis and writing. KX contributed to the sampling, experimental design, data analysis, and writing of the manuscript. All authors contributed to the article and approved the submitted version.

## Conflict of Interest

The authors declare that the research was conducted in the absence of any commercial or financial relationships that could be construed as a potential conflict of interest.

## Publisher’s Note

All claims expressed in this article are solely those of the authors and do not necessarily represent those of their affiliated organizations, or those of the publisher, the editors and the reviewers. Any product that may be evaluated in this article, or claim that may be made by its manufacturer, is not guaranteed or endorsed by the publisher.

## References

[B1] Bao S. D. (2000). *Soil and Agricultural Chemistry Analysis.* Beijing: China Agriculture Press.

[B2] Baptistella J. L. C. de Andrade S. A. L. Favarin J. L. Mazzafera P. (2020). Urochloa in tropical agroecosystems. *Front. Sustain. Food Syst.* 4:119.

[B3] Bolyen E. Rideout J. R. Dillon M. R. Bokulich N. A. Abnet C. C. Al-Ghalith G. A. (2019). Reproducible, interactive, scalable and extensible microbiome data science using QIIME 2. *Nat. Biotechnol.* 37 852–857. 10.1038/s41587-019-0209-9 31341288 PMC7015180

[B4] Bonner M. T. L. Allen D. E. Brackin R. Smith T. E. Lewis T. Shoo L. P. (2020). Tropical rainforest restoration plantations are slow to restore the soil biological and organic carbon characteristics of old growth rainforest. *Microb. Ecol.* 79 432–442. 10.1007/s00248-019-01414-7 31372686 PMC7033081

[B5] Callahan B. J. McMurdie P. J. Rosen M. J. Han A. W. Johnson A. J. A. Holmes S. P. (2016). DADA2: high-resolution sample inference from Illumina amplicon data. *Nat. Methods* 13 581–583. 10.1038/NMETH.3869 27214047 PMC4927377

[B6] Chen H. J. Peng W. X. Du H. Song T. Q. Zeng F. P. Wang F. (2020). Effect of different grain for green approaches on soil bacterial community in a karst region. *Front. Microbiol.* 11:577242. 10.3389/fmicb.2020.577242 33193195 PMC7662124

[B7] Chen Y. L. Xu T. L. Veresoglou S. D. Hu H. W. Hao Z. P. Hu Y. J. (2017). Plant diversity represents the prevalent determinant of soil fungal community structure across temperate grasslands in Northern China. *Soil Biol. Biochem.* 110 12–21. 10.1016/j.soilbio.2017.02.015

[B8] Chi Y. K. Xiong K. N. Xiao H. Chen H. Song S. Z. Shen X. Y. (2020). Study on the relationship between disposition models of forest and grass and soil properties in karst rocky desertification areas of Southwest China. *Fresen. Environ. Bull.* 29 5424–5431.

[B9] Connell R. K. Zeglin L. H. Blair J. M. (2021). Plant legacies and soil microbial community dynamics control soil respiration. *Soil. Biol. Biochem.* 160:108350. 10.1016/j.soilbio.2021.108350

[B10] Crowther T. W. Van den Hoogen J. Wan J. Mayes M. A. Keiser A. D. Mo L. (2019). The global soil community and its influence on biogeochemistry. *Science* 365:eaav0550. 10.1126/science.aav0550 31439761

[B11] Dai Y. T. Yan Z. J. Xie J. H. Wu H. X. Xu L. B. Hou X. Y. (2017). Soil bacteria diversity in rhizosphere under two types of vegetation restoration based on high throughput sequencing. *Acta Pedol. Sin.* 54 735–748. 10.11766/trxb201603150062

[B12] Donald L. G. (1980). Chemical equilibria in Soils. *Clays Clay Minerals* 28 319–319. 10.1346/CCMN.1980.0280411

[B13] Dong L. B. Li J. W. Zhang Y. Bing M. Y. Liu Y. L. Wu J. Z. (2022). Effects of vegetation restoration types on soil nutrients and soil erodibility regulated by slope positions on the Loess Plateau. *J. Environ. Manag.* 302:113985. 10.1016/j.jenvman.2021.113985 34700089

[B14] Edgar R. C. (2013). UPARSE: highly accurate OTU sequences from microbial amplicon reads. *Nat. Methods* 10:996. 10.1038/NMETH.2604 23955772

[B15] Fan Z. Z. Lu S. Y. Liu S. Guo H. Wang T. Zhou J. X. (2019). Changes in plant rhizosphere microbial communities under different vegetation restoration patterns in karst and non-karst ecosystems. *Sci. Rep.* 9:8761. 10.1038/s41598-019-44985-8 31217455 PMC6584648

[B16] Fierer N. Bradford M. A. Jackson R. B. (2007). Toward an ecological classification of soil bacteria. *Ecology* 88 1354–1364. 10.1890/05-1839 17601128

[B17] Ford D. C. Williams P. (2007). *Karst Hydrogeology and Geomorphology*. New York, NY: John Wiley & Sons Ltd.

[B18] Friedman J. Higgins L. M. Gore J. (2017). Community structure follows simple assembly rules in microbial microcosms. *Nat. Ecol. Evol.* 1:0109. 10.1038/s41559-017-0109 28812687

[B19] Gao X. F. Han G. D. Zhang G. G. (2017). Soil microbial community structure and composition of Stipa Breviflora on the desert steppe. *Acta Ecol. Sin.* 37 5129–5136. 10.5846/stxb201605100902

[B20] Griffiths R. I. Thomson B. C. James P. Bell T. Bailey M. Whiteley A. S. (2011). The bacterial biogeography of British soils. *Environ. Microbiol.* 13 1642–1654. 10.1111/j.1462-2920.2011.02480.x 21507180

[B21] Grigera G. Oesterheld M. (2021). Variability of radiation use efficiency in mixed pastures under varying resource availability, defoliation and time scale. *Grassl. Sci.* 67 156–166. 10.1111/grs.12302

[B22] Guerra V. A. Beule L. Mackowiak C. L. Dubeux J. C. B. Blount A. R. S. Wang X. B. (2022). Soil bacterial community response to rhizoma peanut incorporation into Florida pastures. *J. Environ. Qual.* 51 55–65. 10.1002/jeq2.20307 34978336

[B23] Guo Z. Zhang X. Green S. M. Dungait J. A. J. Wen X. Quine T. A. (2019). Soil enzyme activity and stoichiometry along a gradient of vegetation restoration at the karst critical zone observatory in Southwest China. *Land Degrad. Dev.* 30 1916–1927. 10.1002/ldr.3389

[B24] Hartman W. H. Richardson C. J. Vilgalys R. Bruland G. L. (2008). Environmental and anthropogenic controls over bacterial communities in wetland soils. *Proc. Natl. Acad. Sci. U.S.A.* 105 17842–17847. 10.1073/pnas.0808254105 19004771 PMC2584698

[B25] Hu N. Lan J. C. (2020). Impact of vegetation restoration on soil organic carbon stocks and aggregates in a karst rocky desertification area in Southwest China. *J. Soil Sediment.* 20 1264–1275. 10.1007/s11368-019-02532-y

[B26] Hu Y. Wang S. Zeng D. (2006). Effects of single Chinese fir and mixed leaf litters on soil chemical, microbial properties and soil enzyme activities. *Plant Soil* 282 379–386. 10.1007/s11104-006-0004-5

[B27] Jiang Z. C. Lian Y. Q. Qin X. Q. (2014). Rocky desertification in Southwest China: impacts, causes, and restoration. *Earth Sci. Rev.* 132 1–12. 10.1016/j.earscirev.2014.01.005

[B28] Kan H. M. Pang Z. Chen C. Zou J. L. Zhang G. F. Wu J. Y. (2022). Changes in soil microbial communities following the vegetation restoration of degraded sandy grassland in Beijing. *Acta Agrestia Sin.*

[B29] Khatiwada B. Acharya S. N. Larney F. J. Lupwayi N. Z. Smith E. G. Islam M. A. (2020). Benefits of mixed grass-legume pastures and pasture rejuvenation using bloat-free legumes in western Canada: a review. *Can. J. Plant Sci.* 100 463–476. 10.1139/cjps-2019-0212

[B30] Li C. Y. Li X. L. Yang Y. W. Li H. L. Liang D. F. (2020). Effect of nitrogen addition on soil bacterial diversity in alpine degraded grasslands of differing slope. *Acta Pratacult. Sin.* 29 161–170. 10.11686/cyxb2020109

[B31] Li X. K. Su Z. M. Lv S. H. Ou Z. L. Xiang W. S. Qu Z. (2003). The spatial pattern of natural vegetation in the karst regions of Guangxi and the ecological signality for ecosystem rehabilitation and reconstruction. *J. Mt. Sci.* 21 129–139. 10.3969/j.issn.1008-2786.2003.02.001

[B32] Li Y. Liu X. M. Yin Z. Y. Chen H. Cai X. L. Xie Y. H. (2021). Changes in soil microbial communities from exposed rocks to arboreal rhizosphere during vegetation succession in a karst mountainous ecosystem. *J. Plant Interact.* 16 550–563. 10.1080/17429145.2021.2002955

[B33] Lin F. Liu X. J. Zhang J. Y. (2019). Study on the contents of carbon, nitrogen and enzymes activities of sandy soil grown alfalfa and perennial ryegrass with different planting patterns. *Grassl. Turf.* 39 43–49. 10.3969/j.issn.1009-5500.2019.03.006

[B34] Lin X. G. (2010). *Principles and Methods of Soil Microbiology Research.* Beijing: Higher Education Press.

[B35] Ling N. Wang T. T. Kuzyakov Y. (2022). Rhizosphere bacteriome structure and functions. *Nat. Commun.* 13:836. 10.1038/s41467-022-28448-9 35149704 PMC8837802

[B36] Ling N. Zhu C. Xue C. Chen H. Duan Y. Peng C. (2016). Insight into how organic amendments can shape the soil microbiome in long-term field experiments as revealed by network analysis. *Soil Biol. Biochem.* 99 137–149. 10.1016/j.soilbio.2016.05.005

[B37] Liu M. Bai X. Y. Tan Q. Luo G. J. Zhao C. W. Wu L. H. (2021). Monitoring impacts of ecological engineering on ecosystem services with geospatial techniques in karst areas of SW China. *Geocarto. Int.* 10 1–25. 10.1080/10106049.2021.1903580

[B38] Lu Z. X. Wang P. Ou H. B. Wei S. X. Wu L. C. Jiang Y. (2022). Effects of different vegetation restoration on soil nutrients, enzyme activities, and microbial communities in degraded karst landscapes in southwest China. *Forest Ecol. Manag.* 508:120002. 10.1016/j.foreco.2022.120002

[B39] Mi Y. D. Fang H. D. Tao P. Zhou M. Li X. R. Wang F. F. (2021). Comparation study of soil genetic diversity of bacteria and fungi in different vegetation successions in a karst of Guizhou Province, China. *Acta Carsol.* 50 329–342. 10.3986/ac.v50i2-3.7684 17326625

[B40] Momesso L. Crusciol C. A. C. Leite M. F. A. Bossolani J. W. Kuramae E. E. (2022). Forage grasses steer soil nitrogen processes, microbial populations, and microbiome composition in a long-term tropical agriculture system. *Agric. Ecosyst. Environ.* 323:107688. 10.1016/j.agee.2021.107688

[B41] Morrien E. Hannula S. E. Snoek L. B. Helmsing N. R. Zweers H. de Hollander M. D. (2017). Soil networks become more connected and take up more carbon as nature restoration progresses. *Nat. Commun.* 8:14349. 10.1038/ncomms14349 28176768 PMC5309817

[B42] Pan H. Liu H. Y. Liu Y. W. Zhang Q. C. Luo Y. Liu X. M. (2018). Understanding the relationships between grazing intensity and the distribution of nitrifying communities in grassland soils. *Sci. Total. Environ.* 634 1157–1164. 10.1016/j.scitotenv.2018.04.117 29660872

[B43] Pang D. B. Cao J. H. Dan X. Q. Guan Y. H. Peng X. W. Cui M. (2018). Recovery approach affects soil quality in fragile karst ecosystems of southwest China: implications for vegetation restoration. *Ecol. Eng.* 123 151–160. 10.1016/j.ecoleng.2018.09.001

[B44] Paudel S. Cobb A. B. Boughton E. H. Spiegal S. Boughton R. K. Silveira M. L. (2021). A framework for sustainable management of ecosystem services and disservices in perennial grassland agroecosystems. *Ecosphere* 12:e03837. 10.1002/ecs2.3837

[B45] Peng F. Xue X. Li C. Y. Lai C. M. Sun J. Tsubo M. (2020). Plant community of alpine steppe shows stronger association with soil properties than alpine meadow alongside degradation. *Sci. Total. Environ.* 733:139048. 10.1016/j.scitotenv.2020.139048 32446054

[B46] Peng X. D. Dai Q. H. Ding G. J. Shi D. M. Li C. L. (2020). Impact of vegetation restoration on soil properties in near-surface fissures located in karst rocky desertification regions. *Soil Till. Res.* 200:104620. 10.1016/j.still.2020.104620

[B47] Qi D. Wieneke X. Tao J. Zhou X. Desilva U. (2018). Soil pH is the primary factor correlating with soil microbiome in karst rocky desertification regions in the Wushan County, Chongqing China. *Front. Microbiol.* 9:1027. 10.3389/fmicb.2018.01027 29896164 PMC5987757

[B48] Qi X. K. Wang K. L. Zhang C. H. (2013). Effectiveness of ecological restoration projects in a karst region of southwest China assessed using vegetation succession mapping. *Ecol. Eng.* 54 245–253. 10.1016/j.ecoleng.2013.01.002

[B49] Qiao Y. N. Jiang Y. J. Zhang C. Y. (2021). Contribution of karst ecological restoration engineering to vegetation greening in southwest China during recent decade. *Ecol. Indic.* 121:107081. 10.1016/j.ecolind.2020.107081

[B50] Quast C. Pruesse E. Yilmaz P. Gerken J. Schweer T. Yarza P. (2013). The SILVA ribosomal RNA gene database project: improved data processing and web-based tools. *Nucleic Acids Res.* 41 D590–D596. 10.1093/nar/gks1219 23193283 PMC3531112

[B51] Saleem M. (2015). *Microbiome Community Ecology.* Berlin: Springer International Publishing.

[B52] Sansupa C. Wahdan S. F. M. Hossen S. Disayathanoowat T. Wubet T. Purahong W. (2021). Can we use functional annotation of Prokaryotic Taxa (FAPROTAX) to assign the ecological functions of soil bacteria? *Appl. Sci. Basel* 11:688. 10.3390/app11020688

[B53] Segata N. Izard J. Waldron L. Gevers D. Miropolsky L. Huttenhower G. C. (2011). Metagenomic biomarker discovery and explanation. *Genome Biol.* 12:r60. 10.1186/gb-2011-12-6-r60 21702898 PMC3218848

[B54] Shao Y. Q. Ao X. L. Song G. B. Liu R. F. Li H. (2005). Soil microbial biomass in degenerated and recovered grasslands of Huangfuchuan watershed. *Chin. J. Ecol.* 24 578–584.

[B55] Singh J. S. (2015). Microbes: the chief ecological engineers in reinstating equilibrium in degraded ecosystems. *Agric. Ecosyst. Environ.* 203 80–82. 10.1016/j.agee.2015.01.026

[B56] Sui X. Zhang R. Frey B. Yang L. Li M. H. Ni H. (2019). Land use change effects on diversity of soil bacterial, acidobacterial and fungal communities in wetlands of the Sanjiang plain, Northeastern China. *Sci. Rep.* 9:18535. 10.1038/s41598-019-55063-4 31811224 PMC6898332

[B57] Sun F. H. Li X. L. Jin L. Q. Zhao Y. R. Li C. Y. Zhang J. (2022). Changes of soil bacterial community diversity at degraded patches of alpine meadow in the source area of the yellow river. *Environ. Sci.* 43 1–15. 10.13227/j.hjkx.202112106 36096607

[B58] Tang J. Tang X. X. Qin Y. M. He Q. S. Yi Y. Ji Z. L. (2019). Karst rocky desertification progress: soil calcium as a possible driving force. *Sci. Total Environ.* 649 1250–1259. 10.1016/j.scitotenv.2018.08.242 30308895

[B59] Tripathi B. M. Kim M. Singh D. Lee-Cruz L. Lai-Hoe A. Ainuddin A. N. (2012). Tropical soil bacterial communities in Malaysia: pH dominates in the equatorial tropics too. *Microb. Ecol.* 64 474–484. 10.1007/s00248-012-0028-8 22395784

[B60] Wang G. Z. Liu Y. G. Cui M. Zhou Z. Y. Zhang Q. Li Y. J. (2021). Effects of secondary succession on soil fungal and bacterial compositions and diversities in a Karst area. *Plant Soil* 465 1–12. 10.1007/s11104-021-05016-6

[B61] Wang Z. Q. Zhang J. X. Yang X. L. Huang X. Chen S. L. Qiao Y. M. (2021). Characteristics of soil microbial diversity in different patches of alpine meadow. *Acta Agrestia Sin.* 29 1916–1926.

[B62] Wang K. L. Yue Y. M. Chen H. S. Zeng F. P. (2020). Mechanisms and realization pathways for integration of scientific poverty alleviation and ecosystem services enhancement. *Bull. Chin. Acad. Sci.* 35 1264–1272. 10.16418/j.issn.1000-3045.20200729002

[B63] Wang K. L. Zhang C. H. Chen H. S. Yue Y. M. Zhang W. Zhang M. Y. (2019). Karst landscapes of China: patterns, ecosystem processes and services. *Landscape Ecol.* 34 2743–2763. 10.1007/s10980-019-00912-w

[B64] Wang S. J. (2003). The most serious eco-geologically environmental problem in southwestern China – karst rocky desertification. *Bull. Mineral. Petrol Geochem.* 22, 120–126. 10.3969/j.issn.1007-2802.2003.02.007

[B65] Wu Q. Long J. Li J. Liao H. Liu L. Wu J. (2019). Effects of different microhabitat types on soil microbial community composition in the Maolan karst forest in Southwest China. *Acta Ecol. Sin.* 39 1009–1018. 10.5846/stxb201801110084

[B66] Wu W. X. Zhang L. Huang X. Q. Yang X. X. Xue H. L. Liu Y. (2019). Characteristics of soil microbial diversity in different patches of alpine meadow. *Acta Agrestia Sin.* 28 29–41.

[B67] Xie L. W. Zhong J. Cao F. X. Li J. J. Wu L. C. (2015). Evaluation of soil fertility in the succession of karst rocky desertification using principal component analysis. *Solid Earth* 6 3333–3359. 10.5194/se-6-515-2015

[B68] Xing Y. F. Wang X. L. Liu Y. Q. Hua R. Wang C. Wu J. L. (2020). Characteristics of soil microbial diversity in different patches of alpine meadow. *Acta Agrestia Sin.* 28 521–528.

[B69] Xiong K. N. Chi Y. K. Shen X. Y. (2017). Research on photosynthetic leguminous forage in the karst rocky desertification regions of southwestern China. *Pol. J. Environ. Stud.* 26 2319–2329. 10.15244/pjoes/69472

[B70] Xiong K. N. Li P. Zhou Z. F. An Y. L. Lv T. Lan A. J. (2002). *Remote Sensing of Karst Rocky Desertification: A Typical GIS Study—with a special reference to Guizhou Province.* Beijing: Beijing Geological Press.

[B71] Xue K. Zhang B. Zhou S. T. Ran Q. W. Tang L. Che R. X. (2019). Soil microbial communities in alpine grasslands on the Tibetan Plateau and their influencing factors. *Chin. Sci. Bull.* 64 2915–2927. 10.1360/TB-2019-0090

[B72] Xue L. Ren H. D. Li S. Leng X. H. Yao X. H. (2017). Soil bacterial community structure and co-occurrence pattern during vegetation restoration in karst rocky desertification area. *Front. Microbiol.* 8:2377. 10.3389/fmicb.2017.02377 29250053 PMC5717032

[B73] Yang L. Y. Barnard R. Kuzyakov Y. Tian J. (2021). Bacterial communities drive the resistance of soil multifunctionality to land-use change in karst soils. *Eur. J. Soil Biol.* 104:103313. 10.1016/j.ejsobi.2021.103313

[B74] Yang W. T. Wang X. W. Wang J. W. (2013). Crop-and soil nitrogen in legume-gramineae intercropping system: research progress. *Chin. J. Ecol.* 32 2380–2484. 10.13292/j.1000-4890.2013.0342

[B75] Yin R. Deng H. Wang H. Zhang B. (2014). Vegetation type affects soil enzyme activities and microbial functional diversity following re-vegetation of a severely eroded red soil in sub-tropical China. *Catena* 115 96–103. 10.1016/j.catena.2013.11.015

[B76] Young S. D. van Koten C. Gray C. W. Cavanagh J. A. E. Wakelin S. A. (2019). Symbiosis between *Rhizobium leguminosarum* bv. trifolii strain TA1 and a white clover cultivar benefits clover tolerance to cadmium toxicity. *N. Z. J. Agric. Res.* 63 353–364. 10.1080/00288233.2019.1680394

[B77] Yuan D. X. (1991). *Karst of China.* Beijing: Geological Publishing House.

[B78] Yun Y. Wang H. Man B. Xiang X. Zhou J. Qiu X. (2016). The relationship between ph and bacterial communities in a single karst ecosystem and its implication for soil acidification. *Front. Microbiol.* 7:1955. 10.3389/fmicb.2016.01955 28018299 PMC5159436

[B79] Zeng Q. An S. Liu Y. (2017). Soil bacterial community response to vegetation succession after fencing in the grassland of China. *Sci. Total Environ.* 609 2–10. 10.1016/j.scitotenv.2017.07.102 28732294

[B80] Zhang J. Yang X. Song Y. Liu H. Wang G. Xue S. (2020). Revealing the nutrient limitation and cycling for microbes under forest management practices in the Loess Plateau – ecological stoichiometry. *Geoderma* 361:114108.

[B81] Zhang J. L. Wang J. Y. Meng Z. X. He J. Dong Z. H. Liu K. Q. (2022). Soil microbial richness predicts ecosystem multifunctionality through co-occurrence network complexity in alpine meadow. *Acta Ecol. Sin.* 42 2542–2558. 10.5846/stxb202108162255

[B82] Zhang J. X. Wang Z. Q. Quan X. L. Liang J. Shi H. L. Chen M. C. (2021). Responses of soil microbial communities of sown perennial grassland in alpine region to different sowing ways and growth years. *Acta Agrestia Sin.* 29 270–280. 10.11733/j.issn.1007-0435.2021.02.008

[B83] Zhang J. Y. Yang X. M. Song Y. H. Liu H. F. Wang G. L. Xue S. (2019). Revealing the nutrient limitation and cycling for microbes under forest management practices in the Loess Plateau–ecological stoichiometry. *Geoderma* 361:114108.

[B84] Zhao C. Long J. Liao H. K. Zheng C. L. Li J. Liu L. F. (2019). Dynamics of soil microbial communities following vegetation succession in a karst mountain ecosystem, Southwest China. *Sci. Rep.* 9:2160. 10.1038/s41598-018-36886-z 30770852 PMC6377603

[B85] Zhao F. Ren C. Han X. Yang G. Wang J. Doughty R. (2019). Trends in soil microbial communities in afforestation ecosystem modulated by aggradation phase. *For. Ecol. Manag.* 441 167–175. 10.1016/j.foreco.2019.03.036

[B86] Zhu L. Li Y. Yang W. Q. Gao Y. H. (2021). Effect of desertification on soil carbon and nitrogen, enzyme activity and bacterial diversity in alpine grassland. *J. Soil Water Conserv.* 35 350–358. 10.13870/j.cnki.stbcxb.2021.03.048

[B87] Zhu L. F. Liu Y. J. Zhang Q. Jiang C. Jian X. M. Shui W. (2022). Effects of ecological restoration patterns on soil microbial community functional diversity in Zoige alpine desertification grassland. *J. Environ. Eng. Technol.* 12 199–206. 10.12153/j.issn.1674-991X.20210138

[B88] Zi H. B. Liu M. Adi L. J. Hu L. Wang C. T. (2017). Effects of cultivation duration on soil microbial functional diversity of artificial grassland in the Three-River Headwater Region. *Chin. J. Ecol.* 36 978–987. 10.13292/j.1000-4890.201704.003

