# Supplementary material for: Effect of Cultivated Pastures on Soil Bacterial Communities in the Karst Rocky Desertification Area
Source: Front Microbiol. 2022 Jul 28;13:922989. doi: 10.3389/fmicb.2022.922989 (PMC9368201; doi:10.3389/fmicb.2022.922989)
Supplement: Supplementary file 1 [file Table_1.docx]

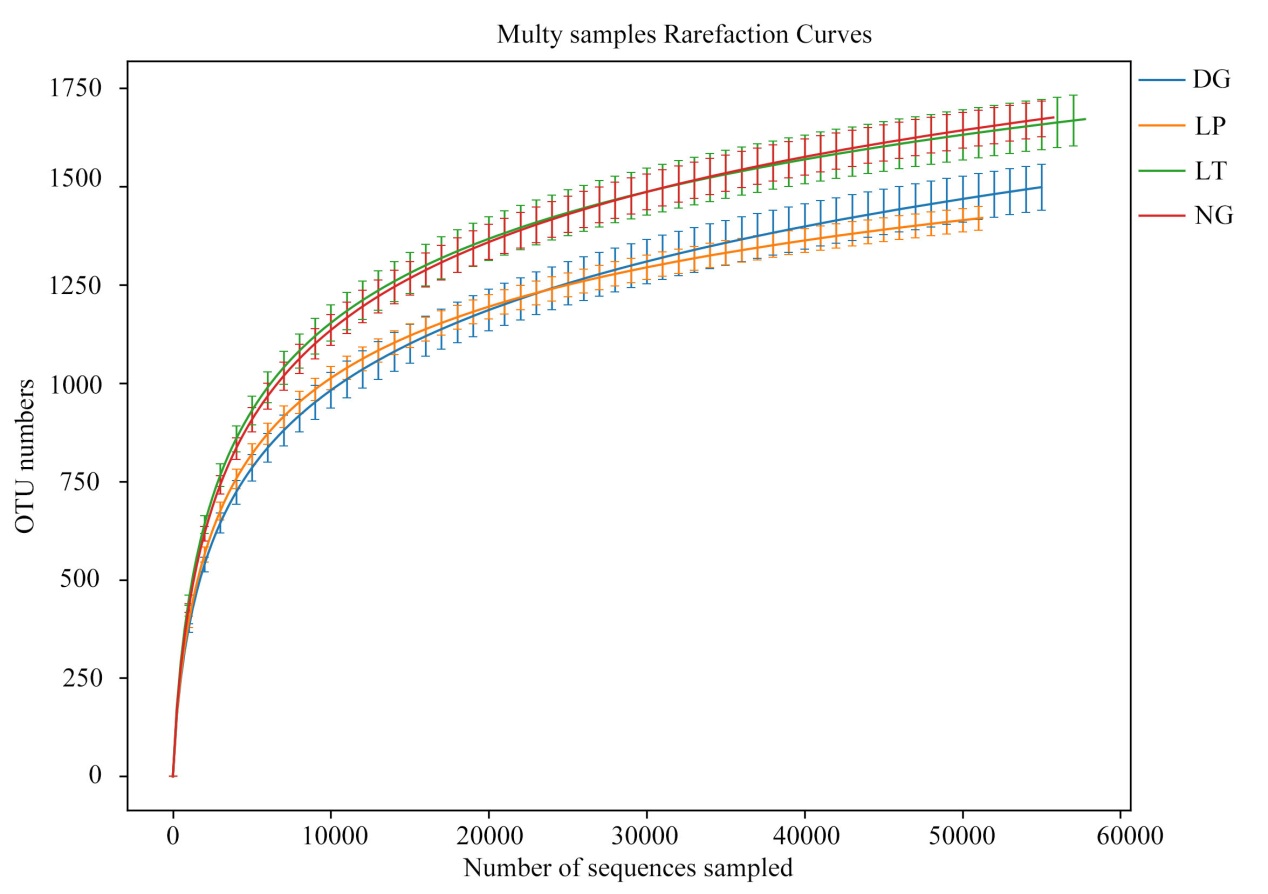


**FIGURE S1 |** OTU quantity rarefaction curve in different grassland vegetation restoration. DG: *Dactylis glomerata*; LP: *Lolium perenne*; LT: *Trifolium repens*; NG: Natural grassland.


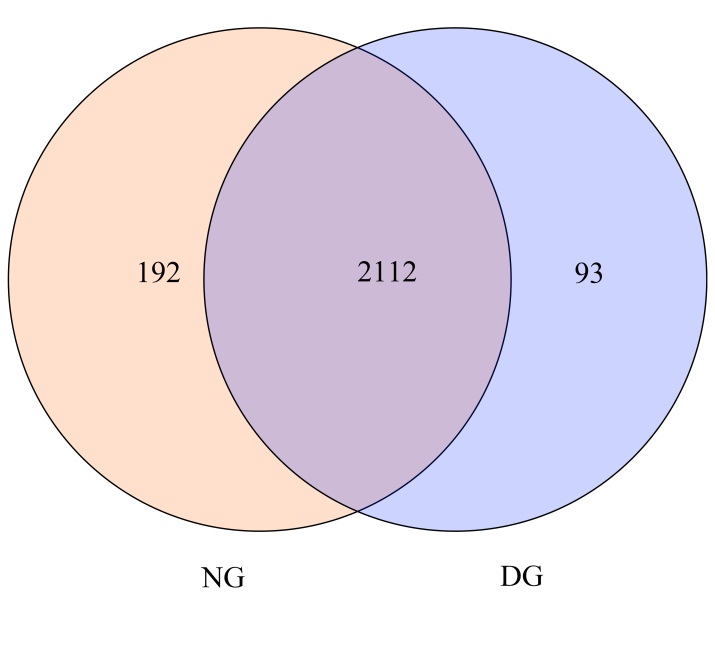


**FIGURE S2 |** Venn diagram of unique and shared bacterial OTUs (at the 3% evolutionary distance) in DG and NG treatment. DG: *Dactylis glomerata*; NG: Natural grassland.


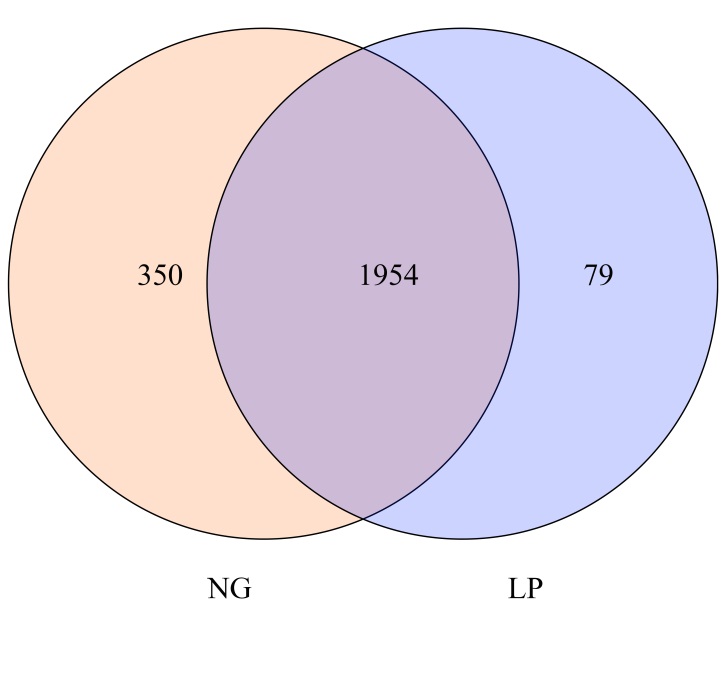


**FIGURE S3 |** Venn diagram of unique and shared bacterial OTUs (at the 3% evolutionary distance) in LP and NG treatment. LP: *Lolium perenne*; NG: Natural grassland.


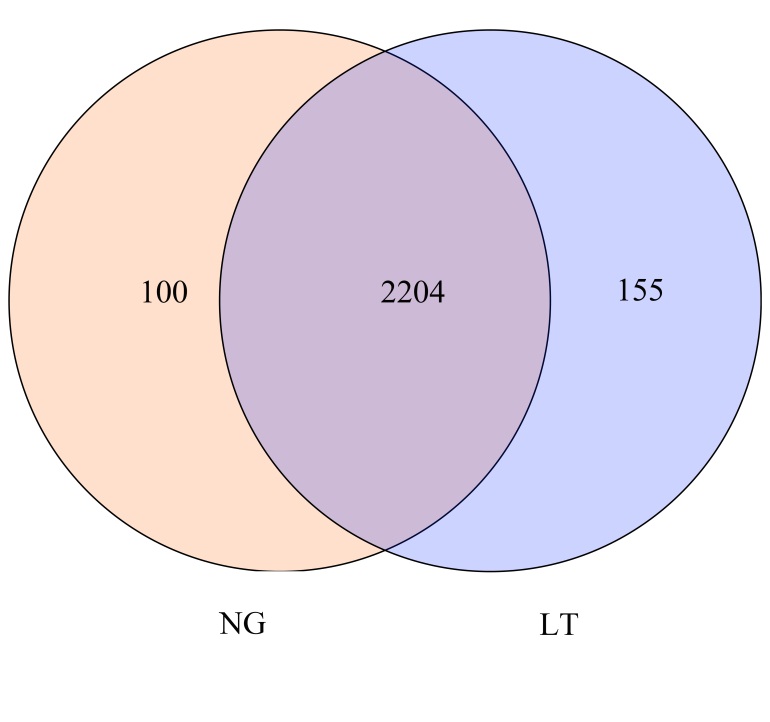


**FIGURE S4 |** Venn diagram of unique and shared bacterial OTUs (at the 3% evolutionary distance) in LT and NG treatment. LT: *Trifolium repens*; NG: Natural grassland.

**TABLE S1** **|** Statistics of sequencing data of Bacterial in different grassland vegetation restoration

| Sample | Raw Reads | Clean Reads | Average Length(bp) |
| --- | --- | --- | --- |
| DG1 | 80088 | 79624 | 415 |
| DG2 | 80270 | 79779 | 416 |
| DG3 | 79707 | 79240 | 415 |
| DG4 | 80134 | 79709 | 414 |
| DG5 | 80164 | 79746 | 413 |
| DG6 | 80339 | 79905 | 415 |
| LP1 | 79861 | 79462 | 415 |
| LP2 | 79664 | 79249 | 417 |
| LP3 | 80113 | 79702 | 416 |
| LP4 | 80074 | 79629 | 417 |
| LP5 | 79899 | 79508 | 416 |
| LP6 | 79777 | 79344 | 416 |
| LT1 | 79804 | 79377 | 419 |
| LT2 | 79917 | 79494 | 418 |
| LT3 | 80274 | 79836 | 421 |
| LT4 | 79600 | 79172 | 418 |
| LT5 | 80499 | 80076 | 417 |
| LT6 | 79715 | 79349 | 416 |
| NG1 | 80221 | 79806 | 416 |
| NG2 | 79572 | 79180 | 417 |
| NG3 | 80108 | 79662 | 416 |
| NG4 | 80026 | 79571 | 418 |
| NG5 | 80046 | 79628 | 416 |
| NG6 | 79812 | 79411 | 415 |

Note: DG, *Dactylis glomerata*; LP, *Lolium perenne*; LT, *Trifolium repens*; NG, Natural grassland.
